# Modeling the social drivers of environmental sustainability among Amazonian indigenous lands using Bayesian networks

**DOI:** 10.1371/journal.pone.0297501

**Published:** 2024-01-25

**Authors:** Robert S. Walker, Jonathan Paige

**Affiliations:** Department of Anthropology, University of Missouri, Columbia, Missouri, United States of America; Institute of Geographic Sciences and Natural Resources Research Chinese Academy of Sciences, CHINA

## Abstract

Amazonia is an invaluable global asset for all its ecological and cultural significance. Indigenous peoples and their lands are pivotal in safeguarding this unique biodiversity and mitigating global climate change. Understanding the causal structure behind variation in the degree of environmental conservation across different indigenous lands–each with varying institutional, legal, and socioenvironmental conditions–is an essential source of information in the struggle for long-term sustainable management of Amazonian ecosystems. Here, we use data from the *Instituto Socioambiental* for 361 indigenous lands in the Brazilian Amazon coded for environmental integrity, territorial integrity, legal stability, indigenous governance, and threats due to infrastructure projects. Using Bayesian networks to learn the causal structure amongst these variables reveals two causal pathways leading to environmental integrity. One causal pathway starts with territorial integrity and is mediated by infrastructure projects, while the other is directly from legal stability. Hence, safeguarding indigenous lands from exploitation is best accomplished via legal land rights and stricter enforcement instead of placing the onus on indigenous governance, which is also a direct outcome of legal stability.

## Introduction

As the world’s largest tropical rainforest, Amazonia is a rich and diverse ecosystem home to an immense array of species and human cultural diversity. As a vital carbon sink, it regulates regional and global climate patterns [1], helps maintain global ecological balance [2], and sustains the livelihoods of thousands of indigenous communities [3]. Amazonian conservation is of paramount global importance, and indigenous peoples are integral to achieving tangible conservation objectives through the stewardship and protection of their homelands [4–6]. Understanding variability in conserving indigenous lands is essential for improving sustainable management practices. The destruction of Amazonian ecosystem functioning, primarily through deforestation and burning, not only releases stored carbon into the atmosphere and diminishes the forest’s capacity to absorb future emissions [7] but erases biocultural diversity [8]. Indigenous lands, where traditional practices prioritize the conservation of forests and waterways, aid in maintaining the Amazon’s carbon sequestration potential, thus playing a critical role in global climate regulation and the long-term sustainability of cultures and nature.

Colonization of Amazonia by non-indigenous populations has resulted in the demographic collapses of many indigenous populations [9–11]. The incessant and irreversible threats of large-scale habitat loss via deforestation, burning, and conversion of land to agriculture and pasture potentially paint a bleak future for indigenous populations [12]. Mining remains a salient threat to indigenous groups as most mining requests to the National Mining Agency in Brazil are on indigenous lands [13]. That indigenous societies survive and maintain traditional lifeways is remarkable, given that powerful external forces from logging, mining, poaching, narcotrafficking, and disease continue to pose such direct existential threats [14, 15].

Despite all the ongoing extractive pressures in Amazonia, indigenous lands suffer less environmental degradation than non-protected areas [16]. The better preservation of indigenous lands is due, at least in part, to their legal status and bolstered levels of protection. They now comprise roughly a quarter of Amazonia and have become the world’s most important strategy for slowing biodiversity loss [17] and deforestation [18] because indigenous lands and communities safeguard against external threats, such as illegal logging and land encroachment [19]. By respecting and supporting the rights of indigenous communities to govern their lands, humanity protects the cultural heritage and the ecosystem goods and services that we ultimately all depend upon.

Understanding the interplay between legal recognition, sovereignty, land management, and environmental sustainability is crucial to effectively protecting critical environments [20]. Various governance arrangements have been proposed as more or less effective at managing and protecting environmental resources. These tend to emphasize one or a mixture of top-down, bottom-up, decentralized, or privatized approaches to managing environmental resources as solutions. However, such arrangements and institutional roles must be tailored to particular socio-ecological contexts [21]. Environmental degradation is often tied to a loss of indigenous control over land and interruption of traditional practices. For example, colonization and industrialization on the Pacific Coast of North America interrupted traditional land use practices, disrupting fire regimes and leading to more destructive wildfires exacerbated by climate change [22]. In Hawaii, colonization and industrial cash crop farming interrupted traditional water and land management systems, introduced non-native plants and grasses, and replaced fire-resistant species, resulting in higher risks of damaging wildfires [23]. Strip mining for nickel in New Caledonia, a world biodiversity hotspot, has transformed erosion patterns, vegetation cover, and water availability [24, 25]. These examples represent long-term industrial and agricultural impacts on environments in colonial and post-colonial contexts where marginalized indigenous communities have little control over land management practices, even where those communities may have degrees of independence, territorial rights, and legal recognition.

Amazonia is a prime example of environmental degradation directly related to the loss of indigenous control over land. The recognition and protection of indigenous claims to territories at the national level in Brazil began in the early 20^th^ century with the establishment of the *Serviço de Proteção aos Índios* (SPI). While nominally intended to protect indigenous groups in Brazil and preserve their culture, the SPI would become a lever for exploitation and genocide [26]. The subsequent period saw limited efforts to protect indigenous territorial claims and environments as many massive public works projects were initiated across Amazonia. The new Brazilian Constitution ratified in 1988 established the rights of indigenous groups within Brazil to live on their territories permanently and afforded them exclusive use of those territories. However, enforcement of those rights has been inconsistent. A natural experiment is underway whereby various indigenous lands in Brazil (also known as Indigenous Protected Areas or Indigenous Territories) are subjected to differential levels of external threats [27], with infrastructure threats such as roads, dams, mines, pipelines, and railroads being those best monitored [28]. Likewise, territorial integrity, legal stability, and indigenous governmental institutions vary across indigenous lands.

The varying institutional, legal, and socioenvironmental conditions across indigenous lands raise essential questions of causality, and these, in turn, have important policy implications. Namely, which driving factors lead to desirable outcomes of better environmental integrity? Also, what other variables mediate these relationships or emerge as downstream effects? Answering these questions should improve our understanding of the socio-ecological context and the relative roles of local indigenous governance (bottom-up) and national law and policy (top-down) in influencing environmental integrity.

We expect three main possible results in this study. First, local community norms and institutions may most effectively protect land against extractive mining, logging, and farming practices [20]. In such cases, we expect in the web of causal relationships that community governance would be “upstream” of other factors, like protection at the federal level. In contrast, it could be that federal-level government protection is the primary driver of the protection of the environment. In this instance, it could be that while diverse local institutions and practices may protect land, those could be less effective without federal-level protections. For example, weak federal protections could allow for larger infrastructure projects to be established in areas that spur more development when completed. Finally, a third expectation is that neither local nor federal-level protections strongly affect environmental protection. In this case, we might expect infrastructure development to be the likeliest candidate for what causes the land to be more or less at risk. No doubt, complexities, and multicausality make this a problematic line of inquiry. Fortunately, analytical tools are available to model and tease apart these causal relationships. In this study, we employ Bayesian networks to propose causal relationships amongst governmental protections, local institutions, and the development and degradation of environments.

## Methods

**Fig 1:**
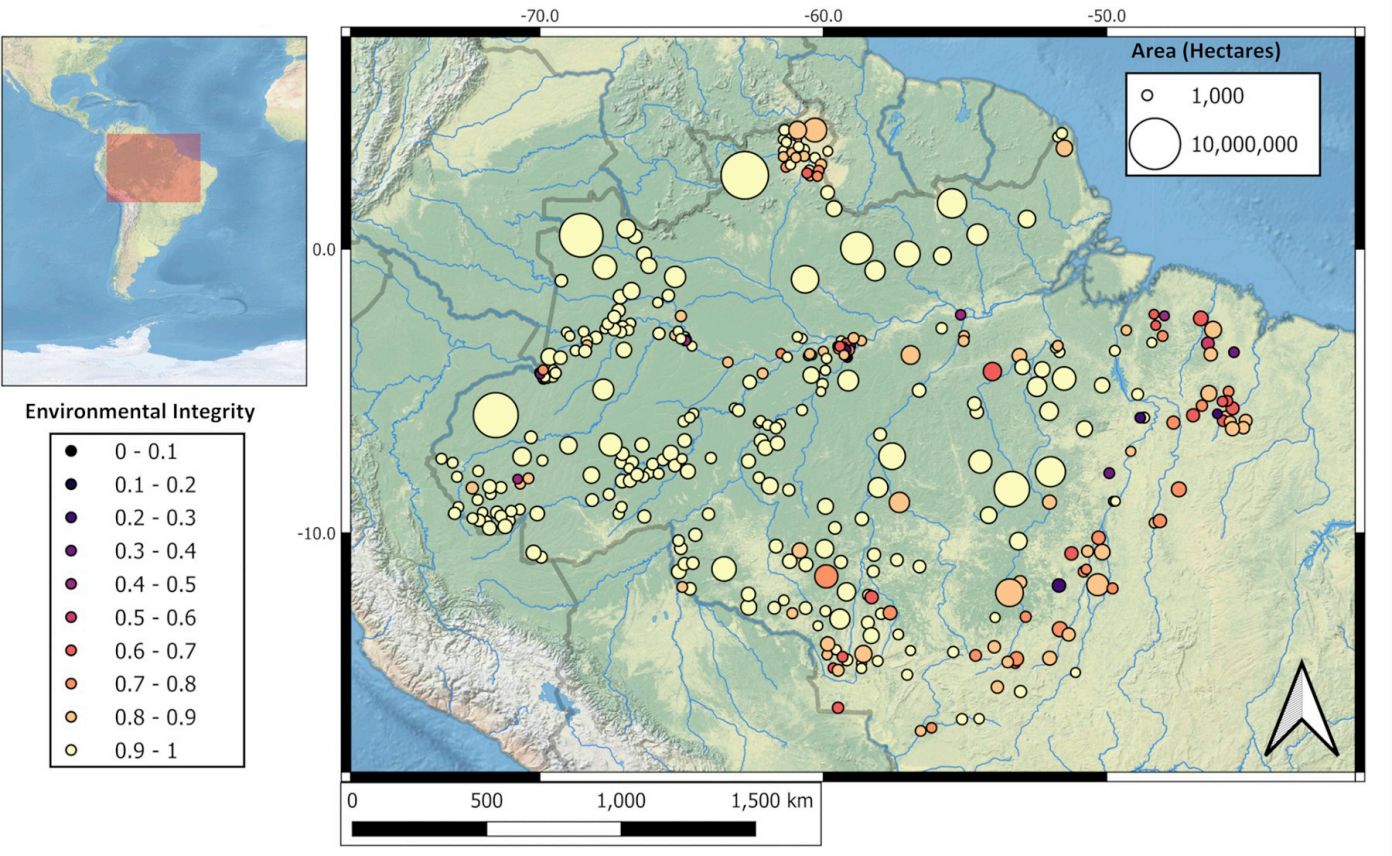
Centroids of Brazilian indigenous lands are colored by environmental integrity and sized according to area. Lands around the southeastern rim of Amazonia, near deforestation, and along the Amazon River tend to score lower on environmental integrity.

The non-profit *Instituto Socioambiental* (https://terrasmais.eco.br) maintains a protected area monitoring program that compiles systematic ratings of different indicators covering institutional, legal, and socioenvironmental conditions for 361 indigenous lands in the Brazilian Amazon (Fig 1). These indicators are quantified by a team of ecologists, biologists, anthropologists, economists, and demographers who consider both primary and secondary data sources that depict the general characteristics of each indigenous land. Indicators are weighted outcomes of multiple inputs. Indicators are better the closer they are to one, with zero representing a worst-case scenario (i.e., min-max normalization).

There are seven available indicators from *Instituto Socioambiental* for each indigenous land. *Environmental integrity* evaluates the integrity of the indigenous land, or how much natural vegetation remains, and its overall resilience to salient threats, namely burning. This is a composite of three inputs: the amount of native vegetation within the territory, the amount of exposure to fires, and the amount of unburned vegetation in the territory. *Environmental integrity in the buffer zone* is the same measure but applied to a 10 km wide buffer zone around the outside of the indigenous land. *Legal stability* relates to the degree of official governmental recognition and legal certainty of land status. This variable represents the degree to which a territory could be under legal or administrative threat. This composite variable incorporates three inputs: the stage at which legal land ownership is recognized at the federal level, whether the registration of the territory has been fully registered at the national level, and whether ongoing legal conflicts are disputing territorial claims. The *absence of threats due to infrastructure projects indicates the current risk due to installed infrastructure projects*, including roads, dams, mines, pipelines, and railroads in or near the indigenous land. The *absence of pressure due to infrastructure projects* indicates the risk due to planned infrastructure projects in the future from all the above projects. *Governance* is the existence of internal mechanisms for indigenous communities to exercise their territorial management. It is composed of four inputs: the reality of formal institutions within communities that serve to manage territories, the existence of territorial and environmental management plans, the presence of projects designed to protect territories, and the absence of territorial overlap with other administrative units. *Territorial integrity* evaluates the degree of security inside the indigenous territory, including exclusive land and resource use rights and the lack of illegal activities and occupation by outsiders. Activities include illegal hunting, fishing, gathering, mining, logging, and other trespassing. Full methodological details for each variable are available online (https://terrasmais.eco.br/doc/nota-tecnica-terrasmais.pdf). Importantly, each variable has strong theoretical linkages to environmental integrity [20, 29–31].

### Bayesian networks

We run Bayesian networks to generate a causal model describing the relationships between legal, social, and environmental variability across Brazilian indigenous territories [32, 33]. Bayesian networks are tremendously valuable tools for producing models describing relationships in complex multivariate datasets standard across the natural [34] and social sciences [35]. Ecologists have used Bayesian networks to understand the causal structure in Amazonian deforestation [36, 37] and to use the networks as decision-making tools [38]. The Bayesian network approach is an alternative to approaches where the dependent variable and independent variables are fixed before analysis, as in path analysis or logistic regressions. Instead, Bayesian networks require no such assumption and algorithmically propose causal structures and their strengths. Bayesian networks evaluate conditional dependencies between variables and submit generative models that could produce those dependencies [39, 40]. Bayesian networks have the advantage, an important one in this case, of not requiring prior assumptions about which variables are predictors, outcomes, or mediators.

As graphical models, Bayesian networks are run in two phases. First, structure learning proposes causal relationships within a matrix of measured random variables (nodes). The directed edges in the graph encode the causal dependencies, while the nodes represent variables. A lack of connection between nodes implies no causal relationship. Second, given the inferred network structure of nodes connected by edges from the first phase, a parameter learning phase then estimates the parameters or coefficients of each network edge. Bayesian networks are directed acyclic graphs, meaning no loops or self-connections are allowed, and causality can only flow in a single direction without feedback. The graphical structure of Bayesian networks allows for modeling direct and indirect causal links, facilitating a deeper understanding of complex systems [41].

We first ran leave-one-out cross-validation tests to evaluate the performance of 11 different Bayesian network algorithms available in the *bnlearn* package [39–42] in *R* version 4.2.2. Of these, the score-based tabu search algorithm outperformed the other options. Selecting tabu as a high-performing algorithm and using the Bayesian Information Criterion to score networks, we generated 10,000 bootstrapped networks by sampling from the original data with replacement. Following the model averaging approach, a robust average network was extracted from these 10,000 bootstrapped networks using a threshold value of 0.8. This value means only relatively strong arcs connecting variables are included in the averaged network. Importantly, even with rather lax threshold values, *environmental integrity* remains an outcome variable, while *territorial integrity* is causally upstream of many other variables.

In addition, the directionalities of edges were computed. These measure the proportion of bootstrapped networks in which edges link together particular nodes in a specific direction. A directionality score of 0.5 indicates the conditional dependencies point one way in 50% of the bootstrapped networks, whereas a directionality score of 1 indicates consistent directionality in 100% of the networks.

### Bayesian path model

In the second phase of the analysis, we used a spatial path model to conduct parameter learning and estimate each network edge’s coefficients, referred to as slopes. This model was implemented in *R* with the *brms* package [43]. We set generic weakly informative priors (mean = 0, sd = 0.1) to the measured slopes and kept the *brms* default priors for the remaining parameters. Because we use indigenous lands as the unit of analysis, this potentially introduces non-independence from spatial clustering [44]. To adjust for the impact of the spatial clustering on our results, we modeled continuous spatial effects with a spline-based 2D smooth term using latitude and longitude. The posterior distributions were sampled using the No-U-Turn Sampler [45] to avoid sampler sensitivity to correlated parameters. The final model was run with four chains for 10,000 iterations, each with a warm-up of 5,000 iterations. For all parameters, r-hat values (a model diagnostic that measures the convergence of models with an expected value equal to one) were exactly one and signify model convergence. Posterior chains were also inspected visually to ensure the No-U-Turn sampler was not becoming “stuck” in particular regions of the parameter space. Using these procedures, we estimated parameter estimates for all the edges in our network. We also generated R-squared values to estimate the total variance explained for each outcome variable in the path model.

## Results

**Fig 2:**
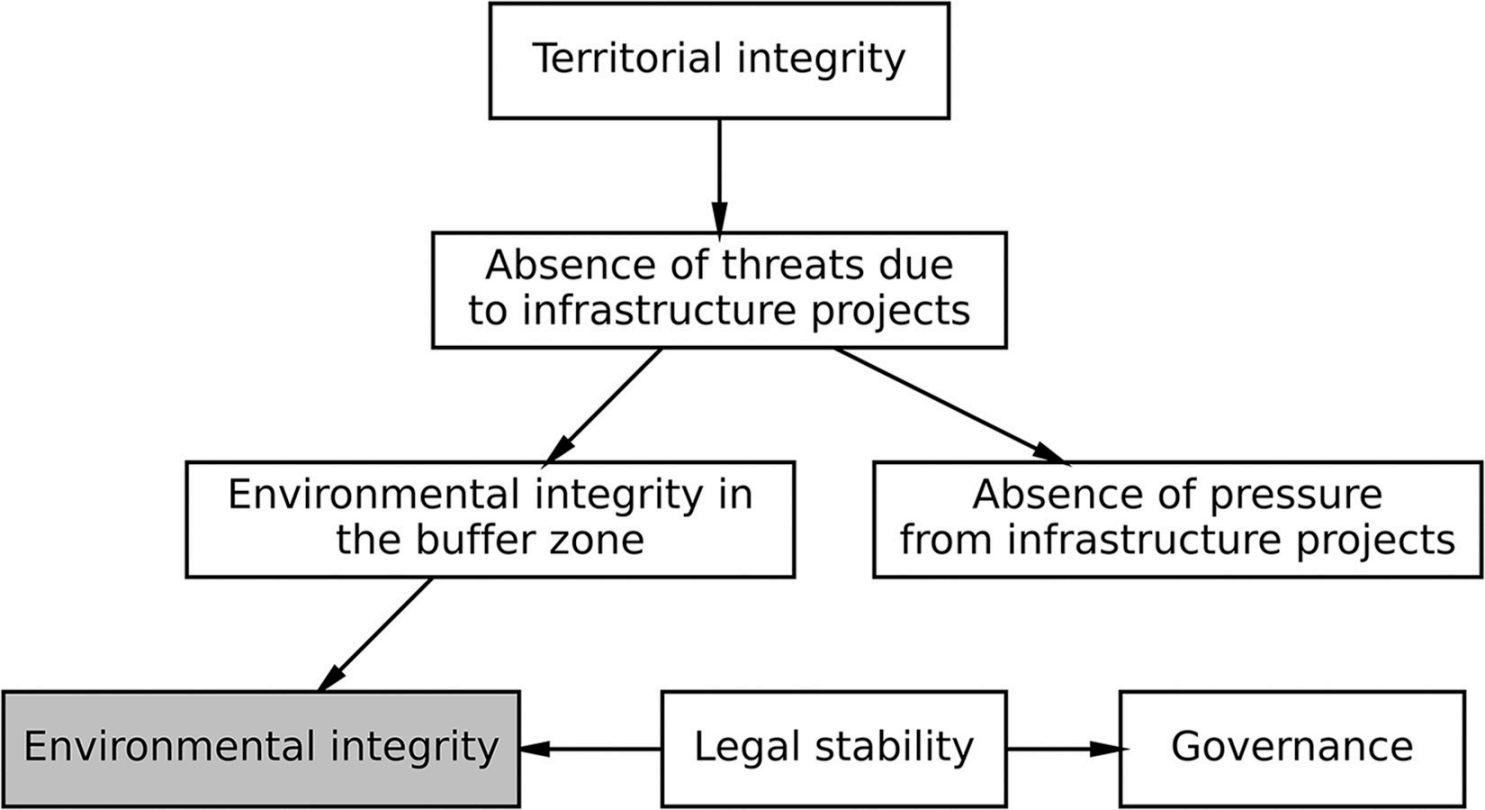
Bayesian network of the seven indicators for Brazilian indigenous lands. There are two main causal pathways to environmental integrity, one originating from territorial integrity and the other directly from legal stability.

Our averaged network based on 10,000 bootstrapped Bayesian networks (Fig 2) found empirical support for six causal relationships that are positive and statistically significant. All these relationships have strong edge strengths ranging from 0.88 to 1 (Table 1), indicating consistent relationships [46]. Edge directionalities in our network range from 0.55 to 0.95, suggesting the model captures mixed evidence for A causing B instead of B driving A. From a conservation perspective, *environmental integrity*, the primary variable of interest, emerges as an outcome variable with two separate causal chains. The first causal chain originates from *territorial integrity* and is mediated by the *absence of threats due to infrastructure projects*. The second causal variable is *legal stability*, which leads directly to *environmental integrity*.

**Table 1:** Bootstrapped Bayesian network results. Network strengths represent the fraction of bootstraps where a link occurs. Directions represent the fraction of bootstraps where links point in that direction. R-squares and slopes (with credible intervals) are from subsequent parameter learning using a spatial path model. All slopes are positive and significant (95% credible intervals do not overlap zero) even when spatial effects are included in the model.

From	To	R-sq	Slope	Strength	Direction
Legal stability	Environmental integrity	0.36	0.10 (0.05–0.16)	0.881	0.905
Legal stability	Governance	0.32	0.25 (0.14–0.36)	0.999	0.918
Environmental integrity in the buffer zone	Environmental integrity	0.36	0.32 (0.25–0.39)	1	0.565
Territorial integrity	Absence of threats due to infrastructure projects	0.32	0.07 (0.01–0.14)	0.917	0.701
Absence of threats due to infrastructure projects	Environmental integrity in the buffer zone	0.43	0.15 (0.06–0.23)	0.898	0.553
Absence of threats due to infrastructure projects	Absence of pressure from infrastructure projects	0.44	0.26 (0.17–0.34)	1	0.945

Another outcome variable that emerges from our network is *governance*. This indicator is indigenous communities’ ability to exercise territorial governance, and it is a result of *legal stability*, like that found for *environmental integrity*. Hence, our network suggests that the legal status of indigenous lands, mainly determined by the Brazilian government, leads to better environmental conservation and effective internal governmental structures of indigenous communities.

The third outcome variable from our network is the *absence of pressure due to infrastructure projects*. These are risks due to planned infrastructure projects in the future, resulting from current infrastructure threats. This implies a chain reaction where existing infrastructure projects lead to additional projects and risks. Conversely, the lack of current infrastructure projects likely acts as a form of protection against future projects. The Bayesian networks correctly capture the causal relationship (i.e., current threats must lead to future pressure and not the reverse) since the directionality is in the correct direction for 95% of the bootstrapped networks.

Bayesian networks are designed so causal relationships are only in one direction without feedback. Yet feedback may be an essential feature of real-world networks. Directionalities provide some information on the potential existence of feedback loops. While most of our directionalities are quite high, there are two edges at 55% and 57%. The first edge with low directionality connects the *absence of threats due to infrastructure projects* and *environmental integrity in the buffer zone*. It is possible that each of these variables affects the other or that both variables index a common factor of encroaching development, or lack thereof. Alternatively, the majority directionality may be causally correct and consistent with the general pattern whereby the creation of infrastructure, namely roads, brings deforestation and further destruction [28]. The second path with a low directionality connects *environmental integrity in the buffer zone* and *environmental integrity*. Again, these variables could be measuring similar phenomena. However, it is also feasible that the majority directionality is indeed in the correct direction, which implies that degradation first tends to start around the outside in the buffer zones and then creeps inwards into the interiors of indigenous lands.

There is considerable spatial clustering in all seven of the indicators we examined. For example, Brazilian indigenous lands around the southeastern rim of Amazonia, near deforestation, and along the main Amazon River tend to score lower on environmental integrity (Fig 1). Generally, this type of spatial clustering is found for all seven indicators, hence the need to adjust for spatial autocorrelation in our results. A spatial model has the effect of weakening some of the relationships found in our Bayesian networks. Still, the primary causal relationships remain positive and statistically significant in our spatial Bayesian path model. Credible intervals (95%) for all six regression slopes describing the relationships between each variable and environmental integrity fall above zero (Table 1).

## Discussion

Our results find that the environmental integrity of indigenous lands emerges from two causal pathways, one originating with territorial integrity and the other from legal stability. Therefore, an important message from these findings is that safeguarding indigenous lands requires a combination of legal land rights and stricter enforcement. According to our networks, indigenous governance directly results from legal stability and is not causally related to environmental integrity. In sum, external forces, instead of internal dynamics, appear primarily responsible for protecting or destroying indigenous lands. While grassroots organizations have their place, indigenous groups can less defend their territorial rights without enforced national laws and policies [47]. Our model pushes for a top-down conservation approach that begins with federal governmental laws and guidelines. Namely, points of weakness do not originate from indigenous governance but from a lack of legal and territorial enforcement at the state and national levels.

Directly related to indigenous governance and territorial integrity are the many indigenous Guardian groups that exist to patrol indigenous lands, limit illegal resource extraction, evict trespassers, and organize road blockades. Examples of Guardians are found amongst the Guajajara, Kayapó, Ka’apor, Munduruku, Uru-Eu-Wau-Wau, Yanomami, and many others. However, the organization of Guardian movements is primarily a response to limited or non-existent amounts of state or national security, as opposed to the driving force behind territorial integrity. In other words, while indigenous organizations and governance can help provide some degree of security inside their territories, maintaining or achieving exclusive land and resource use rights and blocking occupation by outsiders are ultimately the responsibilities of state and national governmental organizations. Our causal analysis of environmental integrity implies that indigenous governance and organization are likely insufficient in the sense that legal land status, recognition, and enforcement, all of which need to be enacted by Brazilian governmental and legal institutions, are necessary to enable or trigger the processes that then lead to the environmental integrity of indigenous lands, namely through the avoidance of infrastructure projects as the key mediating variable.

We do not wish to minimize the multifaceted value and necessity for collaborative efforts to ensure long-term conservation and sustainable management of Amazonian ecosystems. The collective conservation movement should double down on such collaborative efforts. However, from an environmental triage perspective, our results imply that it would be most beneficial for the environmental integrity of indigenous lands for the Brazilian government to increase legal stability for all demarcated indigenous lands, better enforce exclusive resource rights by indigenous occupants, and vigilantly remove illegal activities and occupations by outsiders.

The seven indicators used here are cross-sectional data. Longitudinal data could bolster our claims. While such data are not yet available from *Instituto Socioambiental*, considering areas outside indigenous lands provides longitudinal support for our model. It is reasonable to suppose that most farm and ranch lands in the Brazilian Amazon, most recently forested, would score zeros across the board of indicators used here. Moreover, these unprotected areas, by definition, have no indigenous legal land status and no indigenous territorial integrity, which led to more infrastructure projects and, hence, degraded environmental integrity over time. Therefore, focusing on the worst-case scenario of all zero indicators outside of indigenous lands is a form of longitudinal support for our model, given that these areas are degraded in contrast to indigenous lands [16, 37, 48].

Bayesian networks provide a flexible and intuitive modeling approach to study complex systems with numerous interconnected variables and intricate dependencies. As we see here for this Amazonian natural experiment, the graphical representation of a Bayesian network as a directed acyclic graph enables clear visualization of relationships between variables. It provides a robust methodology for proposing complex causal relationships leading to the environmental integrity of indigenous lands. Interpreting causal structure in the networks helps us gain deeper insights into complex systems and informs decision-making where understanding causality is paramount.

It is well appreciated that indigenous communities nurture profound ancestral connections with their traditional homelands and foster sustainable resource practices. Protecting ecosystem functioning and empowering indigenous communities is a global win-win, requiring more collaboration among governments, non-governmental organizations, and indigenous peoples. Full implementation and respect for indigenous land rights, sustainable development initiatives, and inclusive decision-making processes are essential to successful conservation efforts [49]. Strengthening partnerships that actively engage indigenous communities in forest management with the shared goal of promoting sustainable economic alternatives has the potential to contribute to the long-term preservation of Amazonia [50]. However, protected areas and indigenous lands often lack inadequate funding, limiting environmental protection [51]. Effective conservation in Amazonia demands urgent protection and preservation efforts, with indigenous lands and the communities residing within them playing a central role in conserving biodiversity and mitigating climate change. Recognizing and respecting the rights of indigenous peoples, supporting sustainable land management practices, and fostering collaborative approaches are all important. Yet the causal relationships in our model suggest that the most critical drivers of indigenous land conservation originate with governmental laws and protections and are necessary to ensure the longevity of Amazonia’s irreplaceable ecosystems.
